# Reaction of KHP with excess NaOH or TRIS as standard reactions for calibration of titration calorimeters from 0 to 60 °C

**DOI:** 10.1007/s00249-024-01705-z

**Published:** 2024-04-13

**Authors:** Jason D. Kenealey, Margarida Bastos, Zaid Assaf, Guangyue Bai, Wenqi Zhao, Tyler Jarrard, Colter Tower, Lee D. Hansen

**Affiliations:** 1 Department of Nutrition, Dietetics and Food Science, Brigham Young University, Provo, UT 84602 USA; 2 CIQUP, Institute of Molecular Sciences (IMS), Department of Chemistry and Biochemistry, Faculty of Sciences, University of Porto, Porto, Portugal; 3 Operations Science and Technology, AbbVie, Inc., 1401 Sheridan Road, North Chicago, IL NC-A460064 USA; 4 Collaborative Innovation Center of Henan Province for Green Manufacturing of Fine Chemicals, Key Laboratory of Green Chemical Media and Reactions, Ministry of Education, School of Chemistry and Chemical Engineering, Henan Normal University, Xinxiang, 453007 Henan People’s Republic of China; 5 Department of Chemistry and Biochemistry, Brigham Young University, Provo, UT 84602 USA

**Keywords:** ITC, Potassium biphthalate, Potassium acid phthalate, THAM, Tris(hydroxymethyl)aminomethane, 2-Amino-2-(hydroxymethyl)-1,3-propanediol

## Abstract

Calibration of titration calorimeters is an ongoing problem, particularly with calorimeters with reaction vessel volumes < 10 mL in which an electrical calibration heater is positioned outside the calorimetric vessel. Consequently, a chemical reaction with a known enthalpy change must be used to accurately calibrate these calorimeters. This work proposes the use of standard solutions of potassium acid phthalate (KHP) titrated into solutions of excess sodium hydroxide (NaOH) or excess tris(hydroxymethyl)aminomethane (TRIS) as standard reactions to determine the collective accuracy of the relevant variables in a determination of the molar enthalpy change for a reaction. KHP is readily available in high purity, weighable for easy preparation of solutions with accurately known concentrations, stable in solution, not compromised by side reactions with common contaminants such as atmospheric CO_2_, and non-corrosive to materials used in calorimeter construction. Molar enthalpy changes for these reactions were calculated from 0 to 60 °C from reliable literature data for the p*K*_a_ of KHP, the molar enthalpy change for protonation of TRIS, and the molar enthalpy change for ionization of water. The feasibility of using these reactions as enthalpic standards was tested in several calorimeters; a 50 mL CSC 4300, a 185 μL NanoITC, a 1.4 mL VP-ITC, and a TAM III with 1 mL reaction vessels. The results from the 50 mL CSC 4300, which was accurately calibrated with an electric heater, verified the accuracy of the calculated standard values for the molar enthalpy changes of the proposed reactions.

**Supplementary Information:**

The online version contains supplementary material available at 10.1007/s00249-024-01705-z.

## Introduction

Electrical calibration of calorimeters for measurements of heat is based on the fact that electrical power can be exactly converted into heat and can be measured easily and accurately. However, not all calorimeters can accommodate an electric heater that can be guaranteed to deliver all the heat produced by the heater into the solution in the calorimeter, and therefore to exactly mimic the heat produced by a reaction occurring in the calorimeter vessel. Thus, researchers using titration calorimetry have searched for many years for different chemical reactions that can be used as calibration standards (Velazquez-Campoy et al. 2021; Hansen and Lewis 1971; Sgarlata et al. 2013; Olofsson et al. 2000; Adão et al. 2012; Briggner and Wadsö 1991).

By definition, titration calorimeters titrate a solution of known concentration contained in a motorized syringe into a reactant solution in the reaction vessel. Only power-compensation and heat conduction calorimeters with reaction vessel volumes < 1 mL can meet current demands for use of minimal amounts of reactants, so this work focuses on these two types of solution calorimeters. Errors in measurements of heat with these calorimeters come mainly from four sources: laboratory conditions (e.g., laboratory temperature, temperature variation, air flow, humidity), choice of operating parameters (e.g., equilibration time, injection interval, injection volume, concentrations), data analysis (e.g., peak baselines, blank correction), and calorimeter calibration. Assessing the collective effects of these four sources of error, most of which are dependent on individual circumstances, can only be done with a standard calibration reaction.

**Fig. 1:**
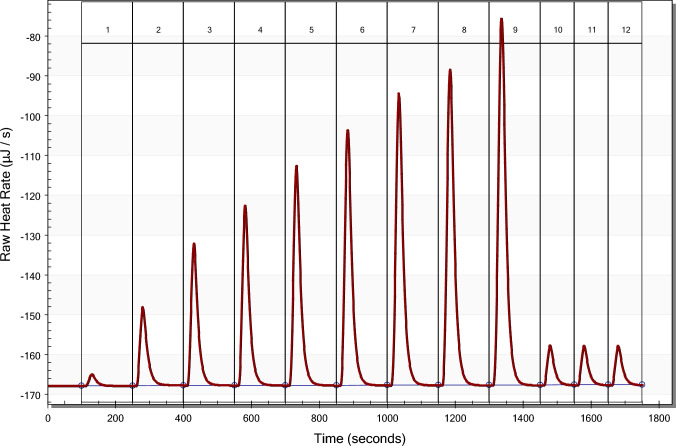
An example of a titration at 25 °C with injections of 1.03, 2.00, 3.02, 3.99, 5.02, 5.99, 7.02, 7.99, 9.98, 1.03, 1.03, and 1.03 μL of 4.97 mM KHP into 185 μL of 49.8 mM NaOH. Note the small area of the first injection as compared with the areas for the last three injections at the same volume. The small area of the first injection is mainly caused by diffusion of titrant from/into the titrant injection tube during the long equilibration time, ≈ 30 min, required to achieve a flat baseline. Note that the time interval between injections is smaller for the last three injections which minimizes titrant diffusion

The output from an incremental titration in an isothermal power-compensation or heat conduction calorimeter is heat rate, dQ/dt, as a function of time, see Fig. 1. Each increment of titrant injected produces a peak in the curve of dQ/dt versus time, and integration of the area under the peak evaluates the total heat from each injection, Q_inj_. Figure 1 shows an example of a titration experiment with increasing injection volumes except for the last 3 injections. The integration baseline for each peak shown in Fig. 1 was set automatically by the NanoAnalyze program, and not further adjusted, as indicated by the lines through the circles superimposed on the baselines. Despite the appearance of high accuracy of baseline placement at the low resolution used in the figure, these baselines should be visually inspected at a higher resolution and corrected before the peaks are integrated.

Evaluating the molar enthalpy change of a reaction in solution (Δ_rxn_*H*) from an incremental titration in a power compensation or heat conduction calorimetry involves the following relations,

1 $${\text{Q}}_{{{\text{rxn}}}} = {\text{c}}_{{{\text{TE}}}} \smallint {\text{s}}\left( {\text{t}} \right){\text{dt}}{-}{\text{b}}$$

where Q_rxn_ is the heat absorbed or released from the reaction that resulted from one injection of titrant into the solution in the calorimetric cell, c_TE_ is the calibration constant relating the measured electrical signal, s(t), to the heat rate, t is time, and b is a blank correction accounting for heat of dilution and/or any side reactions. The integral is taken from the baseline between times t_1_and t_2_, see Fig. 1. Δ_rxn_*H* is then calculated as

2 $$\Delta_{{{\text{rxn}}}} H = {\text{ Q}}_{{{\text{rxn}}}} /\left( {{\text{V}}_{{{\text{inj}}}} {\text{C}}_{{{\text{titrant}}}} } \right)$$

V_inj_ is the volume of titrant injected, C_titrant_ is the concentration of reactant in the titrant solution in moles per liter, and consequently, V_inj_C_titrant_) isthe number of moles of reaction for a quantitative reaction. Obtaining an accurate value for Δ_rxn_*H* by calorimetry thus requires accurate values for five experimental variables, c_TE_,∫s(t)dt, b, V_inj_, and C_titrant_. Or, because some errors may compensate, internally consistent values for the five variables may also provide an accurate value for Δ_rxn_*H*. Note that an electrical calibration only provides a value for c_TE_ while calibration with a standard chemical reaction assesses the collective accuracy of all five variables, as all are involved in the calculation of Δ_rxn_*H*.

ITCs and other titration calorimeters are often used in experiments to simultaneously determine three variables characterizing a binding reaction; stoichiometry, molar enthalpy change, and equilibrium constant. The stoichiometry (usually reported as ‘n’) can only be derived from an experiment if the concentration of the titrant is accurately known, and, if it involves a protein, only if there is certainty that all the protein is fully active. Several reactions have been proposed as test protocols for these determinations, such as the reactions of Ca^2+^ and Mg^2+^ with EDTA in buffers (Velazquez-Campoy et al. 2021; Bastos and Velazquez-Campoy 2021), the reaction of Ba^2+^ with 18-crown-6 (Tellinghuisen 2022), the reaction of HCl with HCO_3_^−^ (Demarse et al 2011), and p-carboxy benzenesulfonamide binding to carbonic anhydrase II (Baranauskiene et al. 2009). In all these cases, the binding isotherm has a well-defined inflection point and therefore the equilibrium constant, *K*, and Δ*H* appear as independent (Velazquez-Campoy 2015). This was also found in a recent study (Velazquez-Campoy et al. 2021) where no significant correlation between *K*, and Δ*H* was found in the study of binding of Ca^2+^ and Mg^2+^ to EDTA. However, ITC was originally developed for determining equilibrium constants for reactions where the binding is too weak to show an inflection point in the binding isotherm (Hansen et al. 1965), a condition that is problematic for most other methods. One of the original papers on the use of titration calorimetry to determine equilibrium constants used the protonation of sulfate ion (p*K* = 1.91) and the reaction of hydroxide ion with monohydrogen phosphate ion (p*K* = 1.61) as standards to test the method. (Christensen et al. 1966) Especially in cases where there is no inflection point, an accurate determination of the thermal calibration constant is required to obtain an accurate value of *K*, because the equilibrium constant and Δ*H* are strongly correlated. In this case, even a constant factor error in Δ*H* causes an error in *K*.

A reaction previously recommended for calibration of the thermal response of solution calorimeters is the reaction of HCl(aq) with tris(hydroxymethyl)aminomethane (TRIS(aq)) (Hansen and Lewis 1971; Sgarlata et al. 2013). However, because HCl(aq) is corrosive to stainless steel, this reagent cannot be used with some current ITCs. Standard solutions of HCl(aq) with an accurately known concentration are difficult to prepare, but can be purchased. TRIS is available in high purity as a weighable solid, but, because the solution is basic, it is prone to absorb CO_2_ and therefore replace reaction with TRIS to reaction with HCO_3_^−^ with a different Δ_rxn_*H*. Further, the most commonly recommended previous procedure with this chemical reaction was to make multiple injections of the same volume of HCl(aq) into a solution of excess TRIS. However, this is not an optimum procedure because the effects of variable heat and injection volumes on the accuracy are not assessed. The procedure proposed in this study, to use a robust test reaction and making titrant injections across the full range of useable volumes of the motorized syringe buret, addresses these questions. A linear relation between heat per injection and injected volume indicates the motorized syringe is delivering the expected volume and that the calorimeter response to the amount of heat is also linear, as expected within a reasonable power/heat range.

Two reactions that avoid the difficulties with the HCl-TRIS reaction, dilution of NaCl(aq) (Tellinghuisen 2007) and heat of mixing of anhydrous n-propanol with water or dilution of 10% n-propanol into water (Olofsson et al. 2000; Adão et al. 2012; Mishina 2022), have also been proposed as chemical standards. Also, Briggner and Wadsö (1991) reviewed chemical calibration of solution calorimeters with Ba^2+^-18-crown-6 at 298, 288 and 310 K, n-propanol dilution at 288, 298, 308, 318, and 348 K, sucrose dilution at 293, 298, 303 and 310 K, and H_2_SO_4_ + HEPES at 298 and 310 K.

The present work proposes two additional calibration reactions with known molar enthalpy changes that can be used to calibrate any titration calorimeter across the full range of temperatures usually accessed by solution calorimetry, i.e., 0–60 °C (273–333 K). The results show the reaction of aqueous potassium acid phthalate (KHP(aq)) with excess NaOH(aq) or with excess TRIS(aq) can be used as standard reactions from 0 to 60 °C for collective calibration of the relevant experimental variables in titration calorimeters. KHP is readily available as a high purity, weighable solid, poses no corrosion problems, and because the basic reactants (NaOH or TRIS) are used in excess, the reactions are not affected by sorption of atmospheric CO_2_. A particular advantage of the proposed reactions is that the molar enthalpy changes are known with reasonable accuracy from the temperature dependence of accurate p*K*_*a*_ values for HP^−^(aq) (Goldberg et al. 2002; Christensen et al. 1976) and from calorimetric measurements on the necessary reactions with a variety of calorimeters (Hansen and Lewis 1971; Goldberg et al. 2002; Christensen et al. 1976; Parker 1965; Olofsson and Hepler 1975).

Thermodynamic values for seven reactions are required for evaluation of the proposed systems for their suitability as standard reactions. The equilibrium constants (as p*K*) and molar enthalpy changes (as Δ_rxn_*H*° with the reaction indicated by the subscript) at 25 °C are given in Eqs. (4–11). Because Δ_rxn_*C*_p_° values are only reliably known at 25 °C, these values are not used in the calculations in this paper, but are included here as an indication of the temperature dependence of Δ_rxn_*H*°. Thus, the Δ_rxn_*H* values and their temperature dependence are determined from the temperature dependence of the equilibrium constants. The values for the phthalate and TRIS acid reactions, 4, 5, and 7–10, are based on data from Goldberg et al. (2002). The values for the water reaction, 6, are from Parker (1965). Note that the value for water ionization, probably the most calorimetrically measured solution reaction of all time, is only known with an accuracy of ± 0.1 kJ mol^−1^ or ± 0.2%.

3 $${\text{H}}_{{2}} {\text{P}}\left( {{\text{aq}}} \right) \, = {\text{ H}}^{ + } \left( {{\text{aq}}} \right) \, + {\text{ HP}}^{ - } \left( {{\text{aq}}} \right){\text{p}}K = { 2}.{95}0,\Delta_{{{\text{H2P}}}} H^\circ = \, - {2}.{7}0{\text{ kJ mol}}^{{ - {1}}} ,\Delta_{{{\text{rxn}}}} C_{{\text{p}}}^\circ = \, - {\text{91 J K}}^{{ - {1}}} {\text{mol}}^{{ - {1}}}$$

4 $${\text{HP}}^{ - } \left( {{\text{aq}}} \right) \, = {\text{ H}}^{ + } \left( {{\text{aq}}} \right) \, + {\text{ P}}^{{{2} - }} \left( {{\text{aq}}} \right) {\text{p}}K = { 5}.{4}0{8},\Delta_{{{\text{HP}}}} H^\circ = \, - {2}.{\text{17 kJ mol}}^{{ - {1}}} ,\Delta_{{{\text{rxn}}}} C_{{\text{p}}}^\circ = \, - {\text{295 J K}}^{{ - {1}}} {\text{mol}}^{{ - {1}}}$$

5 $${\text{H}}_{{2}} {\text{O}}\left( {\text{l}} \right) \, = {\text{ H}}^{ + } \left( {{\text{aq}}} \right) \, + {\text{ OH}}^{ - } \left( {{\text{aq}}} \right) {\text{p}}K \approx {14},\Delta_{{{\text{H2O}}}} H^\circ = \, + {55}.{835} \pm 0.{1}0{\text{5 kJ mol}}^{{ - {1}}} ,\Delta_{{{\text{rxn}}}} C_{{\text{p}}}^\circ = \, - {35}.{\text{5 J K}}^{{ - {1}}} {\text{mol}}^{{ - {1}}}$$

6 $${\text{HP}}^{ - } \left( {{\text{aq}}} \right) \, + {\text{ OH}}^{ - } \left( {{\text{aq}}} \right) \, = {\text{ P}}^{{{2} - }} \left( {{\text{aq}}} \right) \, + {\text{ H}}_{{2}} {\text{O}}\left( {\text{l}} \right){\text{ p}}K \approx {8}.{6},\Delta_{{{\text{KHPH2O}}}} H^{ \circ } = - {53}.{\text{67 kJ mol}}^{{ - {1}}} ,\Delta_{{{\text{rxn}}}} C_{{\text{p}}}^\circ = \, - {\text{259 J K}}^{{ - {1}}} {\text{mol}}^{{ - {1}}}$$

7 $${\text{HT}}^{ + } \left( {{\text{aq}}} \right) \, = {\text{ H}}^{ + } \left( {{\text{aq}}} \right) \, + {\text{ T}}\left( {{\text{aq}}} \right)\quad {\text{p}}K = { 8}.0{72},\Delta_{{{\text{TRIS}}}} H^\circ = \, + {47}.{\text{45 kJ mol}}^{{ - {1}}} ,\Delta_{{{\text{rxn}}}} C_{{\text{p}}}^\circ = \, - {\text{59 J K}}^{{ - {1}}} {\text{mol}}^{{ - {1}}}$$

8 $${\text{T}}\left( {{\text{aq}}} \right) \, + {\text{ H}}_{{2}} {\text{O}}\left( {\text{l}} \right) \, = {\text{ HT}}^{ + } \left( {{\text{aq}}} \right) \, + {\text{ OH}}^{ - } \left( {{\text{aq}}} \right) {\text{p}}K \approx {6},\Delta_{{{\text{TRIShyd}}}} H^\circ = { 8}.{\text{39 kJ mol}}^{{ - {1}}}$$

9 $${\text{HP}} - \left( {{\text{aq}}} \right) \, + {\text{ T}}\left( {{\text{aq}}} \right) \, = {\text{ P}}^{{{2} - }} \left( {{\text{aq}}} \right) \, + {\text{ HT}}^{ + } \left( {{\text{aq}}} \right) {\text{p}}K = { 2}.{664},\Delta_{{{\text{KHPTRIS}}}} H^\circ = \, - {45}.{\text{28 kJ mol}}^{{ - {1}}}$$

## Experimental

### Calorimeters

A NanoITC-LV from TA Instruments, Lindon, UT was used in the Kenealey lab at BYU. A TAM-III from TA Instruments, Lindon, UT was used in the Bai lab in China. A VP-ITC from Microcal/Malvern Instruments was used in the Bastos lab in Portugal. The experiments reported here were used to test the appropriateness of the proposed procedures for chemical calibration in these different, low volume instruments. No attempt was made to compare results from different instruments, i.e., by doing a benchmark study, as that would require a totally different experimental design and was not the purpose of this work.

A CSC model 4300 with a 50 mL Dewar (Calorimetry Sciences Corp., Lindon, UT) was used in the Assaf lab in Wisconsin, and since data from this electrically calibrated calorimeter have an expected accuracy better than 1%, these data were used to verify the accuracy of the literature and derived enthalpy change values. A description of the CSC 4300 calorimeter is necessary to explain why it could be used to verify the values of Δ_rxn_*H* for the proposed standard reactions. The large volume allows construction of a heater that can be guaranteed to provide an accurately known amount of heat into the solution in the 50 mL Dewar used as a reaction vessel. The Dewar, buret (precision syringe driven by a stepper motor), and titrant line are submerged in a constant temperature water bath controlled to ± 1 × 10^–5^ K. The design of the specialized Dewar provides a fixed, well-defined boundary between system and surroundings and because of a very thin inner wall has a response time of < 1 s. The software provided with the calorimeter provides a calibration constant from electric heater calibrations in units of joules per kelvin. Temperature changes are measured with a time constant of approximately 0.2 s and a detection limit of approximately ± 1 × 10^–6^ K with a thermistor in an amplifier bridge. (Christensen et al. 1965) With 50 mL of dilute aqueous solution in the Dewar, the heat detection limit is approximately ± (50 cal mL^−1^ K^−1^) (4.184 J cal^−1^)(1 × 10^–6^ K) =  ± 2 × 10^–4^ J =  ± 0.2 mJ. The titrant delivery volume was calibrated by weighing the water delivered in a specified time. Calibrations were done at 25, 35 and 45°C, and the results show a precision of 0.5%. The typical measurement of 8 J used in this study thus has an expected uncertainty of ± 40 mJ or ± 0.5%.

### Materials

Table 1.

**Table 1:** Materials used in this study

Material	Kenealey	Bai	Assaf	Bastos
KHP (mono potassium salt of o-phthalic acid)	VWR 99.95–100.05%	Sigma-Aldrich 99.95–100.05%	VWR 99.97%	Sigma-Aldrich 99.95–100.05%
TRIS (tris(hydroxymethyl) aminomethane	Fisher Scientific ≥ 99.8%	NA	Sigma-Aldrich 99.9%	NA
NaOH	Fisher Scientific ≥ 97.0%	Macklin ≥ 98.0%	Fisher 5 ± 0.05 N	Merck, ACS Reagent, ≥ 97.0%, pellets
HCl	NA	NA	J.T.Baker 1 N (0.997 M assay)	NA
NaCl	Fisher Scientific ≥ 99.0%	Deen ≥ 98%	Supelco ≥ 98%	Sigma-Aldrich ACS Reagent
HNO_3_	Fisher 68.0—70.0%	Luoyang Haohua Chemical Reagent Co. 65–68%	VWR (0.1N)	Merck 0.1 M HNO_3_ (0.1 N) Titripur
H_2_O	Double distilled	Triple-distilled, CO_2_ free	Super Q water (Millipore, Billerica, MA, USA), N_2_ sparged	Milli Q gradient Ultra pure water (Millipore Billerica, MA, USA)

### Procedures

Assaf—CSC 4300: HCl + NaOH reaction: 0.1–0.9 mL of 200 mM HCl was injected into 50 mL of 50 mM NaOH (n = 8). KHP + NaOH reaction: 0.1–0.9 mL of 100 mM KHP was injected into 50 mL of 50 mM NaOH (n = 8). KHP + TRIS reaction: 0.1–0.9 mL 100 mM KHP was injected into 50 mL of 50 mM TRIS (n = 8). HCl + TRIS reaction: 0.9 mL of 200 mM HCl into 40 mL of 50 mM TRIS (n = 3). For all reactions with multiple injection volumes, the slope of the heat generated vs moles titrated was used to evaluate Δ_rxn_*H* (± SEM, n = 8). Blanks for the KHP(aq) reactions were done by injecting titrant into 50 mL of 50 mM NaCl + 1 mM HNO_3_ (± SEM, n = 4).

Bastos—VP-ITC: Determined at 298.15 K, reference power 25 μJ/s, injection sequence 2, 2, 4, 6, 8, 10, 8, 6, 4, 2, 2, 2, 2 μL, time between injections 150 s for the first two 2 μL injections and 200 s for all remaining injections (including last four 2 μL injections) by titrating 5.304 mM KHP into 1.4323 mL of 52.45mM NaOH. Blank (dilution) correction was made by titrating 5.304 mM KHP into 51.59 mM NaCl + 1.000 mM HNO_3_. For each experiment, the value for Q (after correction for dilution) was plotted against the injected volume and a linear regression was adjusted to the experimental points, forcing the intercept to zero. The average of the slope obtained in each experiment was then divided by the number of moles to obtain Δ_rxn_*H* (± SEM, n = 4).

Kenealey—NanoITC-LV: Determined by titrating 4.97 mM KHP(aq) into 185 μL of 50 mM NaOH(aq). The blank (dilution) correction was obtained by titrating 4.97 mM KHP(aq) into 49 mM NaCl + 1 mM HNO_3_. (± SEM, n = 3). The sequence of injections was 1.03, 2.00, 3.02, 3.99, 5.02, 5.99, 7.02, 7.99, 9.98, 1.03, 1.03, and 1.03 μL with a 50 μL syringe driven by a stepper motor. The heat per injection per μL of titrant injected was evaluated from the slope of a plot of Q_inj_ versus V_inj_.

Bai—TAM III with 4 channels and 1 mL reaction vessels: KHP + NaOH reaction: KHP weighed (0.02709 g) and diluted to 25.00 mL in a volumetric flask. 0.9140 mL 52.44 mM NaOH in reaction vessel titrated with 5.306 mM KHP with a Hamilton gastight 500 μL syringe in aliquots of 2 to 10 μL. Three injection sequences were used (1) 2,2,4,6,8,10,10,8,6,4,2,2,2,2μL; (2) 2,2,2,2,4,4,4,4,6,6,6,6,8,8,8,8,6,6,6,6,4,4,4,4,2,2,2,2 μL; (3) 2,4,6,8,10,8,6,4,2,4,6,8,10,2 μL. Blank (dilution) correction was obtained by titrating 5.306 mM KHP into 51.44 mM NaCl + 1.000 mM HNO_3_. (± SEM, n = 15). For each experiment, the value for Q_inj_ (after correction for dilution) was plotted against the injected volume and a linear regression was adjusted to the experimental points, forcing the intercept to zero. The slope of the plot was used to calculate Δ_rxn_*H*.

Nitric acid was added to the blank NaCl solution to suppress ionization of HP^−^(aq). The 1 mM HNO_3_ concentration is a compromise between suppressing ionization and protonating HP^−^(aq). The NaCl is present to match the ionic strength of the NaOH solution and therefore match the heat of dilution, but if corrosion by an acidic halide solution is of concern, KNO_3_ or NaNO_3_ can be used instead of NaCl. Blanks measured at 25 °C were approximately 3 μJ/μL with 5 mM KHP titrant, ≈ 1% of the 300 μJ/μL from the reaction. Other temperatures gave similar results. Blanks expressed as μJ/μL were multiplied by the injection volume and subtracted from the measured heat per injection at each point.

All calculations were done with Excel. The standard error of the slopes obtained when all repeats were taken together was calculated with the linest function, and the function stdev or stdev.s was used to calculate the standard deviation of the mean from the n points used to obtain the mean value from data expressed as Q_inj_/V_inj_.

### Literature values of relevant quantities and derived quantities

Table 2 shows equations for the relevant p*K*_a_ and Δ*H*° values as functions of temperature.

**Table 2:** Properties of reactions of KHP and TRIS as functions of temperature

	Units, temperature range	Notes	References
KHP, potassium acid phthalate (C_8_H_5_O_4_K, MW = 204.222 g mol^−1^) ionization reaction, HP^−^ = H^+^ + P^2−^,			
p*K*_a2_ = 7.8761 × 10^−5^*T*^2^ − 2.8707 × 10^−3^*T* + 5.4310	*T* is in°C, 0–60 °C	a	Goldberg et al. (2002), pp 326–329, 45HAM/ACR
Δ_r_*G*° = 5.0401 × 10^−4^*T*^2^ − 0.19005*T* + 42.729	*T* is in kelvin, Δ_r_*G*° is in kJ mol^−1^, 0–60 °C	b	
Δ_r_*G*°/*T* = -8.1172 × 10^6^*T*^−3^ + 9.4492 × 10^4^*T*^−2^ − 3.6169 × 10^2^*T*^−1^ + 0.55994	*T* is in kelvin, Δ_r_*G*° is in kJ mol^−1^, 0–60 °C	c	
Δ_HP_*H*° = 3x(-8.1172 × 10^6^)*T*^−2^ + 2x(9.4492 × 10^4^)*T*^−1^ − 3.6169 × 10^2^	*T* is in kelvin, Δ_KHP_*H*° is in kJ mol^−1^, 0–60 °C	d	Method in Lewis et al. (revised by Pitzer and Brewer) (1961), p. 165
At 25°C and Ionic strength = 0, p*K*_a2_ = 5.408, Δ_r_*G*° = 30.869 kJ mol^−1^, Δ_HP_*H*° = − 2.17 kJ mol^−1^			Goldberg et al. (2002, pp 326–329
TRIS, tris(hydroxymethyl)aminomethane (C_4_H_11_NO_3_, MW = 121.136 g mol^−1^) ionization reaction, HT^+^ = H^+^ + T			
p*K*_a_ = 1.0819 × 10^−4^*T*^2^ − 9.2749 × 10^−2^*T* + 26.108	*T* is in kelvin, 0–60 °C	e	Goldberg et al. (2002), pp 357–358, 49BAT/PIN3, 61BAT/HAT, 63DAT/GRZ
Δ_r_*G*° = 9.7427 × 10^−5^*T*^2^ − 6.3587 × 10^−2^*T* + 56.381	*T* is in kelvin, Δ_r_*G*° is in kJ mol^−1^, 0–60°C	b	
Δ_r_*G*°/*T* = 2.5739 × 10^3^*T*^−2^ + 3.0400 × 10^1^*T* + 2.3642 × 10^–2^	*T* is in kelvin, Δ_r_G° is in kJ mol^−1^, 0–60 °C	c	
Δ_TRIS_*H*° = 2x(2.5739 × 10^3^)*T*^−1^ + 30.400	*T* is in kelvin, Δ_TRIS_*H*° is in kJ mol^−1^, 0–60 °C	d	Method in Lewis et al. (revised by Pitzer and Brewer) (1961), p. 165
H_2_O, neutralization reaction, H^+^ + OH^−^ = H_2_O			
Δ_H2O_*H*° = − 55.835 + 0.2238(*T*-25) − 0.00192(*T*-25)^2^	*T*is in°C, Δ_H2O_*H*° is in kJ mol^−1^, 0–60 °C		Parker (1965), p. 19, converted from calories to J by multiplying by 4.184 J/cal

a. Calculated from fitting a quadratic equation to a plot of p*K*_a2_ versus *T*/°C

b. Calculated as Δ_r_*G* = (8.3145*T*p*K*_a2_*2.303)/1000

c. Calculated as the cubic fit to a plot of Δ_r_*G*°/*T* against 1/*T*

d. Calculated as the derivative, d(Δ_r_*G*°/*T*)/d(1/*T*)

e. Calculated from fitting a plot of p*K*_a2_ versus *T* to a quadratic equation

In addition to the references listed in Table 2, another paper (Ford et al. 2001) gives data from which the molar enthalpy change for ionization of HP^−^ can be calculated. However, because the values are at 3.5 bar instead of atmospheric pressure, we decided not to use these values in the present paper.

Table 3 gives the relevant p*K*_a_ and Δ*H*° values at 5 °C intervals calculated with the equations in Table 2 for the reactions used, as those values will be necessary to calculate the molar enthalpy changes for the standard reactions proposed in this study.

**Table 3:** Smoothed values of p*K*_a_ and Δ*H*° values at 5 °C intervals from equations in Table 2 compared with literature values

T/°C	KHP ionization reaction.Δ_KHP_*H*° is in kJ mol^−1^			HT^+^ ionization reaction Δ_TRIS_*H*°is in kJ mol^−1^			H_2_O formation reaction Δ_H2O_*H*°is in kJ mol^−1^			
	KHP, p*K*_a2_^a^	KHP, Table 2 eqn.^a^	KHP, Calorimetric^b^	TRIS, p*K*_a_^a^	TRIS, Table 2 eqn.^a^	TRIS, Calorimetric^b^	H_2_O, Parker (1965), eqn. p.19.^c^	H_2_O, Calorimetric^d^	H_2_O, Calorimetric^e^	H_2_O, Olofsson and Hepler (1975)^f^
0	5.431	3.80		8.846	49.25		− 62.63	− 62.34		− 62.79
5	5.419	2.99		8.680	48.91	49.15, 47.86	− 61.08	− 60.96		− 61.12
10	5.410	2.01		8.520	48.58		− 59.62	− 59.63		− 59.61
15	5.406	0.88		8.365	48.27		− 58.27	− 58.37	− 59.13 ± 0.13	− 58.24
20	5.405	− 0.39		8.216	47.96	47.85	− 57.00	− 57.17		− 56.98
25	5.408 (5.408)^b^	− 1.78 (− 2.17)^b^	− 0.3, − 0.84	8.072 (8.072)^b^	47.67 (47.45)^b^	47.44, 45.73, 48.5, 49.75, 47.53, 47.48, 47.66, 47.50, 47.48, 47.36, 47.42	− 55.84	− 56.02	− 56.44 ± 0.68	− 55.82
30	5.416	− 3.27		7.933	47.38		− 54.76	− 54.94		− 54.73
35	5.427	− 4.85		7.801	47.11	46.81	− 53.79	− 53.91	− 54.65 ± 0.43	− 53.70
40	5.442	− 6.52		7.673	46.84		− 52.91	− 52.94		− 52.73
45	5.461	− 8.26		7.551	46.58		− 52.13	− 52.04	− 52.92 ± 0.58	− 51.79
50	5.484	− 10.07		7.434	46.33	46.03	− 51.44	− 51.19		− 50.88
55	5.511	− 11.93		7.323	46.09		− 50.85	− 50.40	− 50.36 ± 0.61	− 49.99
60	5.542	− 13.83		7.217	45.85		− 50.35	− 49.68		− 49.11

^a^Values of p*K*_a2_ for KHPare calculated from those of W. J. Hamer, G. D. Pinching, and S.F. Acree, Res. Natl. Bur. Stand. (U.S.) 35, 539 (1945) as listed in Goldberg et al. (2002), pp 326–329.p*K*_a_ values for TRISare from pp 357–358 in (Goldberg et al. 2002). Δ_KHP_*H*° and Δ_TRIS_*H*° were calculated from the equations in Table 2

^b^From Golberg et al. (2002) for KHP pp 326–329, and for TRIS pp 357–358. Values in parentheses are the values selected by Goldberg et al. (2002). The value of Δ_KHP_*H*° obtained in this study, − 1.78 kJ mol^−1^may differ from the value from Goldberg et al. (2002), − 2.17 kJ mol^−1^, because we used units of molarity (mol L^−1^) instead of molality (mol kg^−1^) for the K values used in the calculation. Because densities of KHP solutions are only available at 25°C, we chose not to convert the values at 25°C for consistency with data at other temperatures. The values of Δ_TRIS_*H*° obtained in this study from the p*K*_a_ values generally agree with reported calorimetric values within < 1%

^c^From equation on page 19 of Parker et al. (1965) which gives values for Δ_H2O_*H*° as a quadratic equation in (*T*-25) where *T* is the temperature in °C. Parker(1965) cites this equation as accurate to ± 0.105 kJ mol^−1^ from 15 to 30°C but the calorimetric values in Table 3 show it can be extended below (to 5°C) and above (to 60°C) these limits with little loss of accuracy

^d^The values in the table were calculated from fitting a quadratic equation to the values at zero ionic strength in Christensen et al. (1976), pp 207–209

^e^The values in this column were determined by Zaid Assaf in a CSC model 4300 calorimeter with a 50 mL Dewar as part of this project (± SEM, n = 8). Calculations were done as explained in the Data analysis section

^f^From Eq. (9) in Olofsson and Hepler (1975)

## Results

### Reaction of KHP with NaOH

The reaction of KHP(aq) with excess NaOH(aq) can be used to calibrate titration calorimeters, but the standard molar enthalpy change must be corrected for the ionization of HP^−^(aq) in the titrant. The HP^−^ion in the titrant is slightly dissociated and when the KHP solution is injected into the NaOH solution, two reactions occur, described by Eq. (10) above.

10 $${\text{HP}}^{ - } \left( {{\text{aq}}} \right) \, + {\text{ OH}}^{ - } \left( {{\text{aq}}} \right) = {\text{ P}}^{{{2} - }} \left( {{\text{aq}}} \right) \, + {\text{ H}}_{{2}} {\text{O}}\left( {\text{l}} \right)$$

And the inverse of Eq. (10) above, that we now call Eq. (11).

11 $${\text{H}}^{ + } \left( {{\text{aq}}} \right) \, + {\text{ OH}}^{ - } \left( {{\text{aq}}} \right) \, = {\text{ H}}_{{2}} {\text{O}}\left( {\text{l}} \right)$$

The effective heat of reaction, Δ_rxn_*H*, for titration of a solution of KHP into a solution of NaOH must take this into account.

The molar enthalpy change for the combined reactions is given by

12 $$\Delta_{{{\text{rxn}}}} H = \, ({1} - \alpha )(\Delta_{{{\text{KHP}}}} H + \Delta_{{{\text{H2O}}}} H) \, + \, \alpha \Delta_{{{\text{H2O}}}} H$$

where α is the fraction of HP^−^ that is dissociated. Evaluation of α can be done with the known pK_a2_ (see Table 3) and the known analytical concentration of KHP, C, in moles L^−1^.

13 $${1}0^{{ - {\text{pKa2}}}} = {\text{ a}}_{{{\text{H}} + }} {\text{a}}_{{{\text{P2}} - }} /{\text{a}}_{{{\text{HP}} - }} = \, \left( {\left[ {{\text{H}}^{ + } } \right]\left[ {{\text{P}}^{{{2} - }} } \right]/\left[ {{\text{HP}}^{ - } } \right]} \right)(\gamma_{{{\text{H}} + }} \gamma_{{{\text{P2}} - }} /\gamma_{{{\text{HP}} - }} ) \, = \, (\alpha {\text{C}})^{{2}} \gamma_{{{\text{P2}} - }} /({1} - \alpha ){\text{C}}$$

Recasting Eq. (14) in the standard quadratic form gives

14 $$\alpha^{{2}} {\text{C }} + \, \alpha {1}0^{{ - {\text{pKa}}}} {-}{ 1}0^{{ - {\text{pKa}}}} = \, 0{\text{ and }}\alpha \, = \, \left\{ { - {1}0^{{ - {\text{pKa}}}} + \, \left[ {\left( {{1}0^{{ - {\text{pKa}}}} } \right)^{{2}} + {\text{ 4C1}}0^{{ - {\text{pKa}}}} } \right]^{{0.{5}}} } \right\}/{\text{2C}}$$

In Eq. (12), the square brackets around chemical species denote the equilibrium concentration, and γ is the activity coefficient. Note that α is a function of both temperature and concentration. At the low concentrations typically used in ITC the Debye-Hückel limiting law can be used to calculate γ_P2-_, i.e.

15 $$ln\mathrm{\gamma P}2- = -4{A}_{\gamma }\sqrt{I}$$

where ${A}_{\gamma }$ is a temperature-dependent constant and I is the ionic strength. ${A}_{\gamma }$ values were taken from Lewis et al. (revised by Pitzer and Brewer) (1961), p. 640.

In Table 4 we thus report Δ_rxn_*H* for the reaction of KHP(aq) with excess NaOH(aq), calculated with Eqs. (12–15) at three concentrations. Note that the NaOH(aq) excess must be sufficient to prevent reaction with any carbonate from absorption of CO_2_. The p*K*_a_, Δ_KHP_*H*°, and Δ_H2O_*H*° values used in the calculation are from Table 3 columns 2, 3 and 8. In the final column we also report Δ_KHPH2O_*H* in kJ mol^−1^ as obtained in the different calorimeters used here (the Data Analysis section explains how these were obtained).

**Table 4:** Δ_rxn_*H* for the reaction of KHP(aq) with excess NaOH(aq) calculated with Eqs. (11–14) at three concentrations. The p*K*_a_, Δ_KHP_*H*°, and Δ_H2O_*H*° values used in the calculation are from Table 3

T/°C	Δ_KHP_*H*°^a^	Δ_H2O_*H*°^b^	Δ_KHPH2O_*H*/kJ mol^−1c^ with corrected α				Δ_KHPH2O_*H*/kJ mol^−1^ Calorimetric
			0.005 M KHP	0.01 M KHP	0.05 M KHP	0.1 M KHP	
0	3.80	− 62.63	− 58.95	− 58.92	− 58.88	− 58.88	
5	2.99	− 61.08	− 58.19	− 58.16	− 58.13	− 58.13	
10	2.01	− 59.62	− 57.67	− 57.66	− 57.64	− 57.64	
15	0.88	− 58.27	− 57.42	− 57.41	− 57.40	− 57.40	− 56.78 ± 0.32^d^
20	− 0.39	− 57.00	− 57.38	− 57.38	− 57.38	− 57.38	− 57.54 $\pm$ 0.52^d^
25	− 1.78	− 55.84	− 57.56	− 57.58	− 57.59	− 57.60	− 58.04 ± 0.30^d^, − 54.5 ± 1.1^e^, − 57.28 ± 0.05^f^
30	− 3.27	− 54.76	− 57.92	− 57.95	− 57.98	− 57.99	− 56.25 ± 0.51^g^
35	− 4.85	− 53.79	− 58.49	− 58.52	− 58.57	− 58.58	− 58.74 ± 0.47^d^, − 55.7 ± 0.8^e^
40	− 6.52	− 52.91	− 59.23	− 59.27	− 59.34	− 59.35	
45	− 8.26	− 52.13	− 60.14	− 60.20	− 60.27	− 60.29	− 59.54 ± 0.29^d^, − 52.5 ± 2.3^e^
50	− 10.07	− 51.44	− 61.21	− 61.28	− 61.37	− 61.39	− 54.0 ± 1.2^e^
55	− 11.93	− 50.85	− 62.43	− 62.51	− 62.62	− 62.64	
60	− 13.83	− 50.35	− 63.79	− 63.88	− 64.00	− 64.02	

^a^From column 3 in Table 3

^b^From column 8 in Table 3

^c^These values are assessed to be accurate within 1–2% from comparison with literature values and values determined with the CSC calorimeter

^d^Determined by Zaid Assaf in a CSC model 4300 calorimeter with a 50 mL Dewar by titrating 100 mM KHP into 50 mM NaOH. Blank corrected by titrating 100 mM KHP into 50 mM NaCl + 1 mM HNO_3_. (± SEM, n = 8)

^e^Determined by Tyler Jarrad and Colter Tower in a NanoITC-LV in the Kenealey lab by titrating 4.97 mM KHP into 185 μL of 50 mM NaOH. Blank corrected by titrating 4.97 mM KHP into 50 mM NaCl + 1 mM HNO_3_. (± SEM, n = 3)

^f^Determined by Margarida Bastos in a VP-ITC by titrating 5.304 mM KHP into 1.4 mL of 52.45 mM NaOH. Blank corrected by titrating 5.304 mM KHP into 51.59 mM NaCl + 1.000 mM HNO_3_. (± SEM, n = 4)

^g^Determined by Guangyue Bai and Wenqi Zhao in a four-cell TAM-III by titrating 5.306 mM KHP into 0.9140 mL of 52.44 mM NaOH. Blank corrected by titrating 5.306 mM KHP into 51.44 mM NaCl + 1.000 mM HNO_3_. (± SEM, n = 15)

### Reaction of KHP with TRIS

The reaction of KHP(aq)with excess TRIS(aq) can also be used for chemical calibration of titration calorimeters after correction for ionization of HP^−^(aq) in the titrant, similar to what was done above for the reaction of KHP with NaOH. In a solution of TRIS, a small amount of the TRIS is initially hydrolyzed to ^+^HT and an equivalent amount of OH^−^ which, because it is a stronger base than TRIS, would react during the first few injections giving the wrong Q_inj_. Therefore, a small amount of strong acid must be added to the TRIS solution prior to the titration (Sgarlata et al. 2013). The objective is to have the TRIS in the buffer region so HP^−^ only reacts with TRIS and not hydroxide ion. Also, note that because of the small value of pK_KHPTRIS_, to obtain a quantitative reaction, the concentration of TRIS in the reaction vessel needs to be 20 to 100 times the final concentration of KHP in the reaction vessel. A large excess is necessary to prevent the possibility of reaction with bicarbonate from absorbed CO_2_. Table 5 gives the enthalpy change for reaction of KHP titrant with TRIS as a function of temperature and concentration.

**Table 5:** p*K*_KHPTRIS_ and Δ_KHPTRIS_*H* for the reaction of KHP(aq) with excess TRIS(aq) at three concentrations. The fraction of HP^−^ ionized in the titrant was calculated with Eqs. (11–14). The p*K*_a_, Δ_KHP_*H*°, and *Δ*_*TRIS*_*H*° values used in the calculation are from Table 3

T/°C	p*K*_KHPTRIS_^a^	Δ_KHP_*H*°^b^	Δ_TRIS_*H*°/kJ mol^−1^	Δ_KHPTRIS_*H*/kJ mol^−1^ With corrected α^b^				Δ_KHPTRIS_*H*/kJ mol^−1^ Calorimetric
				0.005 M KHP	0.01 M KHP	0.05 M KHP	0.1 M KHP	
0	3.415	3.80	− 49.25	− 45.57	− 45.54	− 45.50	− 45.50	
5	3.261	2.99	− 48.91	− 46.02	− 45.99	− 45.96	− 45.96	
10	3.110	2.01	− 48.58	− 46.63	− 46.62	− 46.60	− 46.60	
15	2.959	0.88	− 48.27	− 47.42	− 47.41	− 47.40	− 47.40	− 47.48 ± 0.58^c^
20	2.811	− 0.39	− 47.96	− 48.34	− 48.34	− 48.34	− 48.34	
25	2.664	− 1.78	− 47.67	− 49.39	− 49.4	− 49.42	− 49.43	− 49.81 ± 0.10^c^, − 47.6 ± 0.9^d^
30	2.517	− 3.27	− 47.38	− 50.54	− 50.57	− 50.60	− 50.61	
35	2.374	− 4.85	− 47.11	− 51.81	− 51.84	− 51.89	− 51.90	− 52.72 ± 0.66^c^, − 47.2 ± 0.3^d^
40	2.231	− 6.52	− 46.84	− 53.16	− 53.20	− 53.27	− 53.28	
45	2.090	− 8.26	− 46.58	− 54.59	− 54.65	− 54.72	− 54.74	− 55.84 ± 0.79^c^, − 51.3 ± 2.3^d^
50	1.950	− 10.07	− 46.33	− 56.10	− 56.17	− 56.26	− 56.28	− 50.5 ± 0.2^d^
55	1.812	− 11.93	− 46.09	− 57.67	− 57.75	− 57.86	− 57.88	
60	1.675	− 13.83	− 45.85	− 59.29	− 59.38	− 59.50	− 59.52	

^a^Calculated from data in columns 2 and 5 in Table 3. Note that this p*K*_KHPTRIS_ is too small for determination in most ITCs but may be useful as a reaction for testing larger volume calorimeters with a higher heat detection limit (Hansen et al. 2011)

^b^These values are assessed to be accurate within 1–2% based on comparison with literature data and data collected with the CSC calorimeter

^c^Determined by Zaid Assaf in a CSC model 4300 calorimeter with a 50 mL Dewar by titrating 100 mM KHP into 50 mL of 50 mM TRIS. (± SEM, n = 8)

^d^Determined by Tyler Jarrard and Colter Tower in a NanoITC-LV in the Kenealey lab by titrating 4.97 mM KHP into 185 μL of 50 mM TRIS. Blank corrected by titrating 4.97 mM KHP into 49 mM NaCl + 1 mM HNO_3_. (± SEM, n = 3)

### Uncertainty in the accuracy of Δ_rxn_*H*° values for the standard reactions

Use of KHP as the acid in the proposed acid–base titrations has an advantage because it is a carboxylic acid which has a relatively small enthalpy change for ionization, i.e., Δ_KHP_*H***°** ≪ Δ_H2O_*H***°**. Therefore, errors in the degree of dissociation, α, and in the activity coefficients, i.e., γ_P2-_, have little effect on Δ_rxn_*H***°**. The largest source of uncertainty is surprisingly the uncertainty in Δ_H2O_*H*° at temperatures below 15 °C and above 30 °C. However, there is a surprisingly good agreement between the values calculated with Parker (1965) and those calculated from a smooth fit of data from Christensen et al. (1976). The largest difference between these two sources is at 60 °C where the difference is 0.67 kJ mol^−1^, still only a 1.4% difference. The values obtained by Assaf in this study also substantiate the accuracy of the data from these two sources. The percentage deviation between calculated values and Assaf’s calorimetric values for the reaction of KHP with NaOH is between 0.3 and 1.4% and for the reaction of KHP with TRIS is between 0.2 and 2.0%. These differences are thus indicative of the absolute accuracy of Δ_H2O_*H*° values except at 25 °C where several carefully measured calorimetric values give a value of 55.835 ± 0.105 kJ mol^−1^ (Parker 1965) or 55.815 ±  < 0.1 kJ mol^−1^ (Olofsson and Hepler 1975).

### Data analysis

Figure 1 clearly shows the measured heat from the first injection of 1 μL is low when compared with the last three 1 μL injections in a NanoITC-LV. The same effect is apparent in comparing the first two injections of 2 μL to the last four 2 μL injections in a VP-ITC (see Supplementary Information). However, all Q_inj_ values, including the first, from the TAM III align with the injected volume, see the Supplementary Information. Because of the way the buret is constructed in the TAM III, the tip of the titrant tube is positioned about 5 mm above the solution in the reaction vessel and the buret is thermostated at the same temperature as the reaction vessel during the equilibration period. The titrant tube is lowered into the solution in the reaction vessel 3–5 min before the titration begins. This procedure minimizes the amount of diffusion to/from the titrant tube prior to the first injection. If the heats from first one or two injections are low, the heats measured in these injections should not be used in analyzing the data from a chemical calibration experiment.

**Fig. 2:**
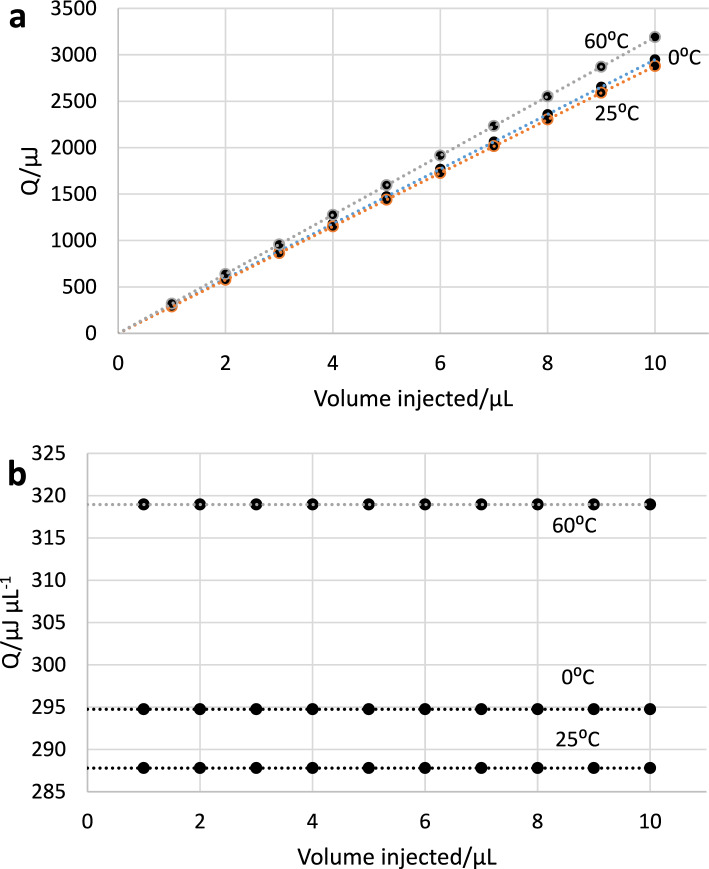
Simulated data using the enthalpy changes for the reaction of KHP with NaOH at 0, 25 and 60 °C with 5 mM KHP titrant to illustrate two ways to view data from an incremental titration with a range of injection volumes. **a** plots the heat per injection versus the volume of titrant injected. **b** plots the heat per injection divided by the volume injected versus the volume injected. The calculated values from the slopes in **a** and from the intercepts in **b** are 294.75, 287.80, and 318.95 μJ μL^−1^ at 0, 25 and 60 °C, respectively. Note that since this is simulated data, there is no error and values for 1 and 2 μL injection are aligned with the other values

Data from a titration such as that in Fig. 1 can be plotted and analyzed in two ways. To illustrate these methods of data analysis, the enthalpy changes for the KHP-NaOH reaction at 0, 25, and 60 °C with 5 mM KHP titrant, see Table 4, were used to generate simulated data. These data are plotted in Fig. 2a, b.

The Supplementary Information shows experimental data from the NanoITC, the VP-ITC, and the TAM III to illustrate the appropriate way to analyze data from the calibrating titrations proposed in this paper. Plots such as those in Fig. 2a emphasize the slope of the plot as determined from a least squares fit of a first order equation to the data (with zero forced intercept) to calibrate the thermal response. However, this plot is a poor way to illustrate random errors among the data points. Figure 2b illustrates the second type of plot, which gives the value of the measured heat per μL injected as the average of the data points as a means of calibrating the thermal response. In both plots, an experimental blank must be subtracted from the data before plotting to correct for the heat of dilution, as outlined above in the Procedures section. If the blank value has been correctly determined and subtracted from the data prior to plotting, and incorrect data, e.g., for first injection volumes < 2 μL, have been removed, the average of the heat per volume should give the same calibration factor as the slope of a linear least squares fit to a plot of heat per injection versus volume, i.e., Fig. 2a.

Examples of the experimental data collected on the KHP-NaOH reaction at 25° with the NanoITC and the VP-ITC and at 30 °C with the TAM III are plotted in accord with Fig. 2a, b in Figs. S1a–S3a and Figs. S1b–S3b in the Supplementary Information. As can be seen in Supplementary Information, the small values for the initial 1–2 μL injections have a larger influence on the average Q/injected volume than on the slope of the plot of Q versus injected volume. Therefore, the initial small injections should not be included in the average value from a plot like 2b to obtain agreement with the slope of a plot like 2a, see Table S1 in Supplementary Information and discussion thereof.

The following procedure is proposed as the optimum way to make use of the data collected on the proposed standard reactions.

1. Perform experiments to determine heat per injection for both the standard reaction and for blanks (dilution heats) as outlined in the Procedures section. At least 3 titrations should be done for each of the standard and the blank, 6 titrations total.
2. Experimental data, corrected for dilution, are then plotted both ways, as heat per injection versus injection volume, i.e. Q_inj_ versus V_inj_ (see Fig. 2a and Supplementary Information Figs. S1a–S3a), and as heat per injection per volume injected versus injection volume, i.e. Q_inj_/V_inj_ versus V_inj_ (see Fig. 2b, and Supplementary Information Figs. S1b–S3b). Add error bars to each plot, equal to twice the standard deviation of the 3 or more replicates used in each plot.
3. Examine the plot of Q_inj_/V_inj_ versus V_inj_ to identify points that are clearly in error as indicated by the error bars and delete these points from the analysis.
4. Force the intercept of the Q_inj_ versus V_inj_ plot to zero since the points with the smallest heat are likely to be the least reliable. Calculate the slope and standard error of the slope (Excel Regression statistics can give this).
5. Calculate the average and standard deviation of the values on the Q_inj_/V_inj_ versus V_inj_ plot.
6. Compare the slope of the Q_inj_/V_inj_ versus V_inj_ with the average of the values on the Q_inj_/V_inj_ versus V_inj_ plot. These values should agree within their combined uncertainties.
7. Calculate the standard value of Q_inj_/V_inj_ from the standard enthalpy change (Table 4 in the case of reaction of KHP(aq) + excess NaOH(aq) and Table 5 for KHP(aq) with excess TRIS(aq) and the concentration of the KHP titrant solution as
  16 $${\text{Q}}_{{{\text{std}}}} ,\mu {\text{J}}\mu {\text{L}}^{{ - {1}}} = \, (\Delta_{{{\text{std}}}} H,\mu {\text{J nmol}}^{{ - {1}}} )({\text{C}}_{{{\text{KHP}}}} ,{\text{ nmol}}\mu {\text{L}}^{{ - {1}}} )$$

A significant disagreement among the three values, i.e. Q_inj_/V_inj_ values in μJ μL^−1^ as obtained from the slope of plot 2a and the average value of plot 2b and the standard value as obtained from Eq. (16) indicates an error in one or more of the parameters, c_TE_, blank, V_inj_, C_KHP_, integration baselines, or another problem that may be external to the calorimeter, e.g. lab temperature. If the two experimental calibration values disagree, the disagreement is likely caused by an error in one or more of the additive factors. If the two experimental calibration values agree, but disagree with the standard value, the disagreement is likely causes by an error in one or more of the multiplicative factors, and the ratio, Q_std_/Q_exp_, can be used as a calibration constant multiplier to correct measured experimental heats. A word of caution about these conclusions; agreement between the two experimental calibration values does not imply that the error is in c_TE_, the error could be in any of the multiplicative parameters.

### Results of calorimetric measurements

**Fig. 3:**
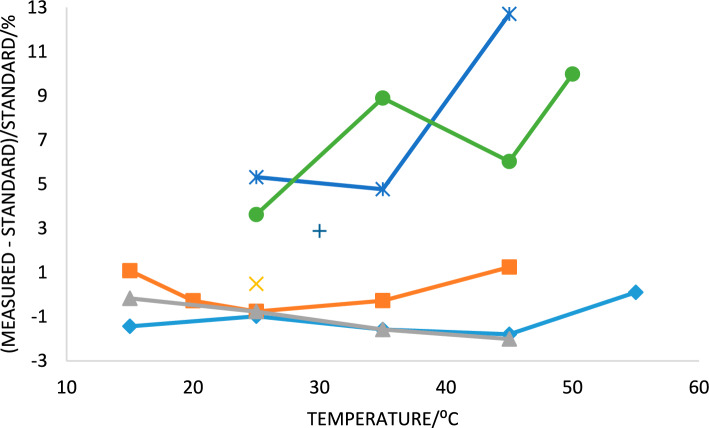
Relative percentage differences [100 × (measured − standard)/standard] between measured Δ_rxn_*H* values and respective standard values given in Tables 3, 4 and 5. Blue with asterisk–Kenealy lab, KHP + NaOH, green with solid circles—Kenealy lab, KHP + TRIS; orange X—Bastos lab, KHP + NaOH; + – Bai lab, KHP + NaOH, red with solid squares—Assaf lab, KHP + NaOH, light blue with diamonds—Assaf lab, HCl + NaOH, gray with filled triangles—Assaf lab, KHP + TRIS. Note that a positive deviation indicates the measured value was less exothermic than the standard

Results from experimental measurements with HCl(aq) as titrant are given in Table 3 and results with KHP titrants are given in Tables 4 and 5. Results were analyzed with both methods shown in Fig. 2a,b as illustrated in Supplementary Information. As expected, the results obtained on all three reactions with the CSC 4300 large volume calorimeter generally show very good agreement with the calculated Δ_rxn_*H* values at all measurement temperatures, and the reactions with NaOH and with TRIS appear to be equally good calibration standards. The measured values are consistent with the expected uncertainties and do not show any consistent deviation with temperature. TAM-III data are only available at 30°C and are a bit less exothermic as compared with the standard value. Several different sequences of injection volumes were tested in the TAM-III and found not to make any significant difference. The VP-ITC data were only collected at 25 °C and are in very good agreement with the standard value. Data were collected with the NanoITC-LV calorimeter for both reactions at temperatures from 25 to 50 °C and were less exothermic than the standards at all temperatures with the deviation from the standards increasing with increasing temperatures. A plot of the deviation of the average values from the standard as determined with the four calorimeters used in this study is given in Fig. 3.

A word of caution is important—these results are for individual calorimeters, conditions, and operators, and are not necessarily representative of all similar calorimeters in different laboratories. Conditions that affect the accuracy differ among laboratories, every calorimeter has its own peculiarities, and every calorimeter ages but not necessarily in the same way, and thus all calorimeters should be regularly and carefully calibrated with a chemical standard. In brief, we do not know why the results with the VP-ITC, NanoITC-LV, and TAM III agreed or did not agree with the standards, and elaboration on this issue would be speculative and is outside the scope of this study. The CSC 4300 is an exception because we know the electrical heater provided an accurate value of the thermal calibration constant and the accuracies of the titrant concentration and buret delivery volume were independently verified (see Methods).

## Discussion

The results of this study demonstrate that KHP titrated into excess NaOH(aq) or TRIS(aq) are effective reactions for collectively testing the calibration of all five experimental variables in titration experiments in low volume ITCs. These reactions have several advantages over the previously proposed standards: (1) KHP is an easily weighable solid, (2) KHP is not corrosive to the stainless steel parts in titration syringes, (3) because the titration is done into excess base, atmospheric CO_2_ does not interfere with the reaction, and (4) the molar enthalpy changes for these reactions are well known over a wide range of temperature, i.e., 0–60 °C.

The previously proposed chemical calibration reactions fit into three classes of reaction; dilution (e.g., NaCl (Tellinghuisen 2007) and propan-1-ol (Oloffson et al. 2000; Adão et al. 2012; Mishina 2022), binding (e.g., Ba^2+^ to 18-crown-6 (Sgarlata et al. 2013; Briggner and Wadsö 1991; Tellinghuisen 2022), Ca^2+^ and Mg^2+^ to EDTA (Velazquez-Campoy et al. 2021; Bastos and Velazquez-Campoy 2021), and p-carboxy benzenesulfonamide to carbonic anhydrase II (Baranauskiene et al. 2009), or acid–base titrations (e.g., HCl into bicarbonate (Demarse et al. 2011) and HCl into TRIS (Hansen and Lewis 1971; Sgarlata et al. 2013). Each of these reactions has advantages and disadvantages. Dilution reactions can only be used for calibrating the thermal calibration constant, injection volumes, and possibly the active cell volume, i.e., NaCl dilution. Enthalpies of dilution are only known at temperatures near room temperature for propan-1-ol, but are known over a wide temperature range for NaCl. The binding and acid–base reactions are clearly appealing because the majority of the reactions run in ITCs are binding reactions with the purpose of simultaneous determination of the equilibrium constant and the molar enthalpy change. These reactions thus collectively test the thermal response, injection volume, and active cell volume of the calorimeter as well as the software modeling. However, these reactions are difficult to use as standards because weighable standards are not readily available for some of the reactants, and the molar enthalpy changes are sensitive to ionic strength and pH. Halide acids are also corrosive to the stainless steel parts in injection syringes used in some ITCs. Equilibrium constants and molar enthalpy changes for these reactions are also not well known except near room temperature.

Use of KHP as the acid in the proposed acid–base titrations has an advantage because it is a carboxylic acid which has a relatively small molar enthalpy change for ionization, i.e., Δ_KHP_*H*° ≪ Δ_H2O_*H*°. Therefore, errors in the degree of dissociation, α, and in the activity coefficients, i.e., γ_P2-_, have little effect on Δ_rxn_*H*°. Also, KHP has long been used as an acidimetric standard, the acid ionization constant is well known as a function of temperature (Goldberg et al. 2002), and is available in high purity from multiple sources. The values obtained by Zaid Assaf in this study generally substantiate the accuracy of the calculated data. Taken together, the three sets of results from the Assaf lab range from + 1 to − 2% from the standards, have an average deviation from the standards of − 0.66%, and a slope of only − 0.0009% per °C. The largest source of uncertainty in the proposed KHP reactions is surprisingly the uncertainty in Δ_H2O_*H*°. However, there is good agreement among the values calculated with the Parker (1965) equation, those calculated from a smooth fit of data from Christensen et al. (1976), and from the Olofsson and Hepler (1975) equation. The largest difference between Parker (1965) and Christensen et al. (1976) is at 60 °C where the difference is 0.67 kJ mol^−1^, only a 1.4% difference. The difference between Parker (1965) and Olofsson and Hepler (1975) is nearly 2.5% at 60 °C. These differences are thus indicative of the absolute accuracy of Δ_H2O_*H*° except at 25 °C where several carefully measured calorimetric values give a value of 55.835 ± 0.105 kJ mol^−1^ (Parker 1965)or 55.815 ±  < 0.1 kJ mol^−1^ (Olofsson and Hepler 1975) which is only ± 0.2%.

## Conclusions

The need for calibration of titration calorimeters with a well-defined standard reaction must always be stressed as it is a critical factor determining the accuracy of reported data, and consequently the agreement among different reports from different labs or different users for the same system. The proposed reactions of KHP(aq) with NaOH(aq) or TRIS(aq) appear to fulfil the requirement for an easy to use and reproducible chemical system that can be used at temperatures from 0 to 60 °C. The Δ_rxn_*H* for these standard reactions is known with an uncertainty in the absolute accuracy of about 1% at temperatures around 25 °C and increasing to about 2% at higher temperatures. By injection of differing volumes covering the entire range used with any given calorimeter, the proposed method collectively tests the consistency of all five experimental variables, c_TE,_ ∫s(t)dt, b, V_inj_, and C_titrant_, as explained in the introduction.

### Supplementary Information

Below is the link to the electronic supplementary material.

Supplementary file1 (DOCX 70 KB)

## Data Availability

All relevant, applicable data are included in the text or supplementary material.

## Abbreviations

*K*: Equilibrium constant
pK_a_: Negative log_10_ of an acid ionization constant
Q: Heat
Q_inj_: Heat absorbed or released as a result of an injection of titrant
t: Time
s: Signal
b: Blank
V_inj_: Volume injected
C: Concentration in moles per liter
T: Temperature in Celsius or Kelvin as specified
KHP: Potassium hydrogen phthalate
TRIS: Tris(hydroxymethyl)aminomethane
^+^HT: Protonated TRIS
Δ_rxn_H: The enthalpy change associated with each reaction, the particular reaction being designated with subscripts as H2P for the first ionization of phthalic acid, HP for the second ionization of phthalic acid, H_2_O for the formation of water by reaction of H^+^(aq) with OH−(aq), KHPH2O for the reaction of HP^−^(aq) with OH^−^(aq), TRIS for the reaction of TRIS(aq) with H^+^(aq), TRIShyd for the reaction of TRIS with H_2_O to form ^+^HT, and KHPTRIS for the reaction of HP^−^(aq) with TRIS(aq).
γ: Activity coefficient
