# Supplementary material for: Reaction of KHP with excess NaOH or TRIS as standard reactions for calibration of titration calorimeters from 0 to 60 °C
Source: Eur Biophys J. 2024 Apr 13;53(4):225–38. doi: 10.1007/s00249-024-01705-z (PMC11098914; doi:10.1007/s00249-024-01705-z)
Supplement: Supplementary file 1 — Supplementary file1 (DOCX 70 KB) [file 249_2024_1705_MOESM1_ESM.docx]

**Supplementary Information (SI)**

Reaction of KHP with excess NaOH or TRIS as standard reactions for calibration of solution calorimeters from 0 to 60°C

Jason D. Kenealey^1^, Margarida Bastos^2^, Zaid Assaf^3^, Guangyue Bai^4^, Wenqi Zhao^4^, Tyler Jarrard^1^, Colter Tower^1^, Lee D. Hansen^5^

1. Department of Nutrition, Dietetics and Food Science, Brigham Young University, Provo, UT 84602 USA.

2.CIQUP, Institute of Molecular Sciences (IMS), Department of Chemistry and Biochemistry, Faculty of Sciences, University of Porto, Porto, Portugal.

3. Operations Science and Technology, AbbVie, Inc.,NC-A4, 1401 Sheridan Road, North Chicago, IL 60064 USA.

4. Collaborative Innovation Center of Henan Province for Green Manufacturing of Fine Chemicals, Key Laboratory of Green Chemical Media and Reactions, Ministry of Education, School of Chemistry and Chemical Engineering, Henan Normal University, Xinxiang, Henan 453007, P. R. China.

5. Department of Chemistry and Biochemistry, Brigham Young University, Provo, UT 84602 USA.

Corresponding author: Lee D. Hansen, ldhansen@chem.byu.edu, Ph 801-422-3667, fax 801-422-0153

The purpose of this Supplementary Information is to illustrate the procedures and data analysis described in the paper with the actual data obtained in this study using three different microcalorimeters and the chemical reactions proposed as standard reactions for calibration of titration calorimeters. The results at 25⁰C or 30 ⁰C where results are available for the three calorimeters are given in this supplementary. The plots shown present the heat per injection and the heat per injection per volume injected, both versus the volume injected (Figures S1a-S3b). The heats of reaction are corrected for the experimentally determined heat of dilution as described in the main text. Note that the intercept is forced to zero in plots of the heat per injection versus the injection volume for the titrations as per the instruction #4 in the main text, and all points are included in the plots. A table collecting the relevant parameters is also provided (Table S1).

**List of Figures and Table**

| **Figure S1a**. Experimental data from Jason Kenealy lab collected with a NanoITC-LV (TA Instruments, Lindon, UT) for the reaction of KHP(aq) with excess NaOH(aq) at 25⁰C, plotting the heat per injection versus the volume of titrant injected. |  | S4 |
| --- | --- | --- |
| **Figure S1b.** Experimental data from Jason Kenealy lab collected with a NanoITC-LV (TA Instruments, Lindon, UT) for the reaction of KHP(aq) with excess NaOH(aq) at 25⁰C, plotting the heat per injection per volume injected versus the volume of titrant injected. |  | S4 |
| **Figure S2a.** Experimental data from Margarida Bastos lab collected with a VP-ITC (Microcal/Malvern Instruments) for the reaction of KHP(aq) with excess NaOH(aq) at 25⁰C, plotting the heat per injection versus the volume of titrant injected. |  | S5 |
| **Figure S2b.** Experimental data from Margarida Bastos lab collected with a VP-ITC (Microcal/Malvern Instruments) for the reaction of KHP(aq) with excess NaOH(aq) at 25⁰C, plotting the heat per injection per volume injected versus the volume of titrant injected. |  | S5 |
| **Figure S3a.** Experimental data from Guangyue Bai lab collected with a TAM-III with 4 channels and 1 mL reaction vessels (TA Instruments, Lindon, UT) for the reaction of KHP(aq) with NaOH(aq) at 30⁰C, plotting the heat per injection versus the volume of titrant injected. |  | S6 |
| **Figure S3b.** Experimental data from Guangyue Bai lab collected with a TAM-III with 4 channels and 1 mL reaction vessels (TA Instruments, Lindon, UT) for the reaction of KHP(aq) with excess NaOH(aq) at 30⁰C, plotting the heat per injection per volume injected versus the volume of titrant injected. |  | S7 |
| **Table S1.** Summary of the results from the plots of experimental data S1a to S3b at 25 and 30⁰C. |  | S8 |

.

Figure S1. Experimental data from Jason Kenealy lab collected with a NanoITC-LV (TA Instruments, Lindon, UT) for the reaction of KHP with NaOH at 25 ⁰C, with 4.97 mM KHP titrant into 50 mM NaOH, using a range of injection volumes injected in the sequence 1.03, 2.00, 3.02, 3.99, 5.02, 5.99, 7.02, 7.99, 9.98, 1.03, 1.03, and 1.03 μL with a 50 μL syringe driven by a stepper motor (n=3). Figure **S1a** plots the heat per injection versus the volume of titrant injected and Figure **S1b** plots the heat per injection divided by the volume injected versus the volume injected. In all cases the error bars represent twice the standard deviation of the repeat experiments. The slope obtained in **S1a** when adjusting a straight line with intercept zero, together with its standard error are provided in Table S1 (2^nd^ and 3^rd^ columns), as well as the mean and standard deviation for the values in **S1b** (4^th^ and 5^th^ columns).

**a**

**b**

Figure S2. Experimental data from Margarida Bastos lab data collected 25⁰C atwith a VP-ITC (Microcal/Malvern Instruments. Experiments were performed using reference power 25 μJ/s, injection sequence 2, 2, 4, 6, 8, 10, 8, 6, 4, 2, 2, 2, 2 μL, time between injections 150 s for the first two 2 μL injections and 200 s for all remaining injections (including last four 2 μL injections), titrating 5.304 mM KHP into 1.4323 mL of 52.45mM NaOH (n=4). Blank correction was made by titrating 5.304 mM KHP into 51.59 mM NaCl + 1.000 mM HNO_3_. Figure **S2a** plots the heat per injection versus the volume of titrant injected and Figure **S2b** plots the heat per injection divided by the volume injected versus the volume injected. In all cases the error bars represent twice the standard deviation of the repeat experiments. The slope obtained in **S2a** when adjusting a straight line with intercept zero, together with its standard error are provided in Table S1 (2^nd^ and 3^rd^ columns), as well as the mean and standard deviation for the values in **S2b** (4^th^ and 5^th^ columns).

**a**

**b**

Figure S3. Experimental data from Guangyue Bai lab collected at 30⁰C with a TAM-III with 4 channels and 1 mL reaction vessels (TA Instruments, Lindon, UT). Experiments were performed by titrating 5.306 mM KHP into 0.9140 mL of a 52.44 mM NaOH solution. A Hamilton gastight 500 μL syringe was used for KHP, with injection volume sequence 2,2,4,6,8,10,10,8,6,4,2,2,2,2 μL (n=5). Blank correction was obtained by titrating 5.306mM KHP into 51.44 mM NaCl + 1.000 mM HNO_3_. Figure **S3a** plots the heat per injection versus the volume of titrant injected and Figure **S3b** plots the heat per injection divided by the volume injected versus the volume injected. In all cases the error bars represent twice the standard deviation of the repeat experiments. The slopes obtained in **S3a** when adjusting a straight line with intercept zero, together with its standard error are provided in Table S1 (2nd and 3rd columns), as well as the mean and standard deviation for the values in **S3b** (4^th^ and 5^th^ columns). Note that the error bars are here smaller than the data points.

**a**

**b**

It is worth noting that the measured heat from the first injection of 1 μL is low when compared with the last three 1 μL injections in a NanoITC-LV, the same effect being apparent when comparing the first two injections of 2 μL to the last four 2 μL injections in a VP-ITC. This effect is not present in the results from TAM III from Bai’s lab, where all Q values align with the injected volume. The reason for the lack of discrepancy in the first injections in this case results from a special procedure used. The titrant tube is not inserted in the solution in the vessel during the equilibration period but is about 5 mm away being thermostated at the correct temperature during this period, but not in contact with the solution in the vessel. When the system reaches equilibrium, the tip of the titrant tube is inserted into the solution in the vessel and the titration starts in 3-5 minutes. This procedure reduces diffusion to/from the needle tip to about the same that occurs in all subsequent injections, and thus the heat released is not diminished for the first injection.

Table S1. Summary of the results from the plots of experimental data in Figures S1a to S3b at 25 and 30 ⁰C.

| Calorimeter | Slope, from “a” plots,  μJ/μL | Std. error of the slope,  μJ/μL | Average, from “b” plots,  μJ/μL | Std. Dev. from “b” plots,  μJ/μL | Standard,  μJ/μL assuming ΔH = -57.56 kJ mol^-1^ |
| --- | --- | --- | --- | --- | --- |
| NanoITC-LV | 273^a^ | ±6 | 238^a^  272^b^ | ±75  ±15 | 286.1 |
| VP-ITC | 304^a^ | ±5 | 287^a^  301^b^ | ±37  ±12 | 305.3 |
| TAM-III^c^ | 298.9^a^ | ±0.4 | 298^a^ | ±3 | 305.6 |

^a^ All points included.

^b^ Excluding first two 2 μL injections.

^c^ at 30^o^C.

The values in Table S1 show that for both the NanoITC-LV and the VP-ITC, the slope of type “a” plots agrees with the average values from type “b” plots within the combined uncertainties if the small, initial injections are not considered in the average. Regarding the TAM III, the values for the slope and the average with all values included are in complete agreement. The influence of the incorrect too small values for the initial 1-2 μL injections is much larger on the average Q/injected volume than on the slope of Q versus injected volume with a forced zero intercept. It is also worth noting that the uncertainty of the average Q/injected volume is larger than the uncertainty in the slope of a type “a” plot, even when the small, initial injections are not considered in the average.

Finally, it should be stressed that a significant disagreement among the three values of the heat per injected volume (μJ/μL), i.e., as obtained from the two plots of the experimental data and the value for the standard under the specified conditions (2^nd^, 4^th^ and 6^th^ columns in Table S1), indicates an error in one or more of the parameters, c_TE_, blank, V_inj_, C_KHP_, integration baselines, or another problem that may be external to the calorimeter, e.g. lab temperature. Variation in any one, some, or all of these factors accounts for the dispersion observed in Figure 3 in the main text, and further, indicates that all ITCs may have individual quirks and should be calibrated with a standard chemical reaction.
